# Systematic pan-cancer analysis identifies PKNOX1 as a potential prognostic and immunological biomarker and its functional validation

**DOI:** 10.3389/fimmu.2025.1533690

**Published:** 2025-06-23

**Authors:** Kan Liu, Yongkang Xu, Ye Mao, Shumin Fu, Shenglan Huang, Jianbing Wu

**Affiliations:** Department of Oncology, The Second Affiliated Hospital, Jiangxi Medcical College, Nanchang University, Nanchang, China

**Keywords:** PKNOX1, pan-cancer, prognostic biomarker, immune infiltration, HCC and breast cancer

## Abstract

**Background:**

PBX/knotted1 homeobox 1 (PKNOX1), also known as PREP1, is a homeodomain transcription factor that has been reported to be involved in the progression of gastric adenocarcinoma and non-small cell lung cancer. However, its expression, clinical significance, and biological function in various human cancers have not been studied in depth, and its role and specific molecular mechanism in the occurrence and development of cancer remain unclear. Its potential as a cancer prognostic marker and therapeutic target has not yet been explored.

**Methods:**

The TCGA and GTEx databases were used to evaluate the differential expression of PKNOX1 pan-cancer, and PKNOX1 gene mutation information was obtained from the cBioPortal and GSCALite platforms. Cox regression and Kaplan-Meier analyses were used to evaluate the value of PKNOX1 in pan-cancer prognosis. The correlations between PKNOX1 expression and the expression of DNA methylation-related genes, immune-related genes, tumor mutation burden (TMB), and microsatellite instability (MSI) were analyzed using Spearman correlation. The correlations between PKNOX1 expression and MDSC immune infiltration and immune cells were analyzed using the TIDE algorithm and the ESTIMATE algorithm. PKNOX1 -interacting proteins and expression-related genes were analysed via the STRING and TIMER 2.0 platforms, and the functions of PKNOX1 in tumors and the cell pathways involved were predicted via KEGG enrichment analysis. In addition, the differential expression and function of PKNOX1 in HCC and breast cancer were explored via Western blotting and proliferation and migration-related experiments.

**Results:**

PKNOX1 is significantly highly expressed in most tumor types and is significantly associated with poor patient prognosis and increased clinicopathological stage. Tumor gene mutations and DNA methylation may explain the abnormal expression of PKNOX1. In most tumors, PKNOX1 expression was significantly correlated with MDSC immune infiltration and immune cells, methylation-related genes, immune-related genes, the TMB and MSI. The enriched KEGG pathways indicated that PKNOX1 is involved in cancer-promoting processes such as cell-cell adhesion, the cell cycle and cell proliferation and deterioration signalling pathways. *In vitro* experiments revealed that PKNOX1 is highly expressed in HCC and breast cancer cells and HCC tissues and promotes the growth, proliferation, migration and invasion of HCC and breast cancer cells.

**Conclusion:**

PKNOX1 is a promising prognostic and immune biomarker in pan-cancer and may play an important role in HCC and breast cancer progression and metastasis.

## Background

Cancer is a malignant disease that is difficult to cure and is associated with a high mortality rate. It is still a major public health problem and a major cause of death worldwide, and has a serious impact on human health and life (1). According to the World Health Organization (WHO), the global incidence and mortality rates of malignant tumors have increased annually. In 2022, there were 19.98 million new cases of malignant tumors worldwide and 9.74 million deaths from tumors. It is estimated that by 2050, the number of cancer cases worldwide will reach 35 million, significantly increasing the global cancer burden (2–4). Although many advances in clinical tumor treatment have been made and immunotherapy and targeted therapy are widely used as promising tumor treatments (5–7), the survival prognosis of patients is still unsatisfactory due to the heterogeneity of tumors and the special immune microenvironment (8, 9). Therefore, identifying specific immune biomarkers and new gene targets for tumor occurrence and development is highly important for providing a theoretical basis for tumor diagnosis, prediction and treatment strategies (10, 11). Owing to the complexity of tumor development, it is very important to conduct pan-cancer expression and functional analyses of potential target genes and evaluate their correlation with clinical prognosis and potential molecular mechanisms.

PKNOX1 (PBX/knotted1homeobox1, also known as PREP1) belongs to the three amino acid loop extension(TALE) superfamily of homeobox proteins. It is a homeodomain transcription factor and a cofactor of Hox proteins and is involved in growth and differentiation during vertebrate embryogenesis (12–14). The TALE subclass includes two families, PBC (PBX1-4) and Meniox, and the Meniox family, which consists of two subfamilies: Meis (Meis1-3) and PREP (PREP1-2) (15). PKNOX1 can achieve and strengthen DNA binding through heterodimerization with other homeodomain partners (PBC family members (PBX1-4)), regulate the subcellular localization and stability of both proteins, and regulate transcription by binding to multiple promoters and interacting with multiple proteins (12, 16). It has been reported that PKNOX1 can promote the progression and metastasis of various tumors, including gastric adenocarcinoma (17) and non-small cell lung cancer (18). However, there is currently no pan-cancer evidence from clinical data or in-depth basic research on the relationships between PKNOX1 and various tumor types. The expression level and clinical significance of PKNOX1 in most tumor types remain unclear, and its biological role remains to be elucidated. Therefore, the use of a pan-cancer database to further study the regulatory function of PKNOX1 in tumors and the molecular mechanism of occurrence and development are highly important.

Our study is the first to use the TCGA and GTEx databases to perform pan-cancer analysis of PKNOX1. The expression patterns and genetic mutation characteristics of PKNOX1 in a human pan-cancer dataset were comprehensively explored, and the relationships between PKNOX1 and pan-cancer survival prognosis and clinical pathology were analysed. The correlations between PKNOX1 expression in pan-cancer, the tumor immune microenvironment and tumor immunity were investigated. The expression pattern and biological function of PKNOX1 in pan-cancer were elucidated from multiple dimensions, highlighting the potential of PKNOX1 as a pan-cancer prognostic and immune marker, and exploring the potential mechanism of PKNOX1 in the occurrence and development of pan-cancer. In addition, we conducted a series of experiments to determine the expression level of PKNOX1 in HCC and breast cancer cell lines and tissues, and explored its role in tumor growth, proliferation, migration and invasion in HCC and breast cancer cells.

## Materials and methods

### Gene expression analysis

Systematic analysis of immune infiltration in different tumor types was performed via Tumor Immune Estimation Resource 2.0 (TIMER2.0, http://timer.cistrome.org). We entered “PKNOX1” in the “Gene_DE” module of the TIMER 2.0 webpage to obtain the differences in PKNOX1 expression between tumors and adjacent normal tissues in 33 cancer types from The Cancer Genome Atlas (TCGA) database. Since some tumor types have no normal tissue or the number of normal tissue cases is small, we entered “PKNOX1” in the “Single Gene Analysis” module in GEPIA2 (Gene Expression Profiling Interactive Analysis, version 2, http://gepia.cancer-pku.cn/index.html) and obtained data from The Cancer Genome Atlas (TCGA) and the Genotype Tissue Expression Database (GTEx) for supplementation.

Protein expression analysis was performed via the UALCAN portal (http://ualcan.path.uab.edu/analysis-prot.html) in the Clinical Proteomic Tumor Analysis Consortium(CPTAC) dataset. We entered “PKNOX1” in the search box to explore its protein expression levels between primary tumors and normal tissues. Six tumor datasets (breast cancer, ovarian cancer, colon cancer, clear cell RCC (renal cell carcinoma), UCEC (uterine corpus endometrial carcinoma), and LUAD (lung adenocarcinoma)datasets) were selected for analysis.

We further explored the specificity of PKNOX1 mRNA expression in different tissues, single cell types, and blood cell lineages. In the HPA (The Human Protein Atlas, https://www.proteinatlas.org/) webpage, “PKNOX1” is entered into the “Tissue”, “Single Cell Type” and “Immune Cell” modules to obtain results directly from the HPA database. Similarly, the “TISSUE and PATHOLOGY” and “SUBCELL” modules were used on the HPA webpage to obtain immunohistochemical images and immunofluorescence localization images of PKNOX1 protein expression, respectively.

### Gene mutation analysis

We used the cBioPortal(https://www.cbioportal.org/) webpage to obtain information on gene variation characteristics. In the “Quick search Beta” of the webpage, we entered “PKNOX1” to obtain the variation characteristics of the PKONX1 gene. In the “Cancer Types Summary” module, we observed the variation frequency, mutation type, and CNA (copy number variation) results of all TCGA tumors. Through the “Mutations” module, the mutation site information, protein structure diagram and 3D (three-dimensional) structure of PKNOX1 were obtained. GSCALite (http://bioinfo.life.hust.edu.cn/web/GSCALite/) is an online tumor gene set analysis platform that integrates tumor genomic data of 33 tumor types from the TCGA. We obtained the associations between PKNOX1 expression and gene copy number variation (CNV) and DNA methylation status through the “Mutation” module of the GSCALite webpage. Because PKNOX1 gene is closely associated with DNA methylation, we further performed Spearman correlation analysis on the basis of gene co-expression in the TCGA database to evaluate the relationship between PKNOX1 expression and the expression of nine methylation-related genes (ALKBH3, ALKBH1, YTHDC1, YTHDF1, YTHDF3, YTHDF2,TRMT6,TRMT10C,TRMT61B, TRMT61A) in 33 tumors. Differences were considered statistically significant when the p value was < 0.05.

### Correlation analysis between PKNOX1 expression and clinical characteristics and prognosis

To investigate the pan-cancer prognostic value of PKNOX1, we first analysed the correlation between PKNOX1 expression and pan-cancer survival prognosis. We downloaded relevant data from the TCGA database, integrated the expression data and clinical information of tumors, deleted incomplete data on survival time and survival status, and then used the “survival” and “survminer” packages of R 4.3.0 to explore the correlations between PKNOX1 expression and pan-cancer median survival (OS), disease-specific survival (DSS) and progression-free interval (PFI) via the Kaplan-Meier method and univariate Cox regression analysis. The best statistical cut-off values ​​were used to divide patients with different types of tumors into high expression groups and low expression groups. The log-rank test and Cox regression were used to calculate the significance of the survival time between the two groups. The prognostic results are expressed as hazard ratios (HR), 95% confidence intervals, and p-values, with p-values < 0.05, indicating statistical significance.

We also analysed the correlations between different clinical characteristics such as tumor size (T stage), tumor grade (G grade), pathological grade, and tumor subtype and PKNOX1 expression levels via the “limma” and “ggpubr” packages of R 4.3.0. The Wilcoxon rank sum test was used to compare gene expression levels according to different clinicopathological characteristics, and a p value < 0.05 was considered statistically significant. Differences in tumor type (p < 0.05) are shown in box plots.

### Correlation of PKNOX1 with the TME and tumor immunity

We used TIMER2 to explore the association between PKNOX1 expression and immune infiltration in all tumors in the TCGA database. By entering “PKNOX1” in the “Gene” module of the “Immune” section of the webpage, we selected myeloid-derived suppressor cells (MDSCs) and used the TIDE algorithm for immune infiltration analysis. P values ​​and partial correlation (cor) values ​​were obtained via a purity-adjusted Spearman rank correlation test. The data are presented in the form of heatmaps and scatter plots. We then downloaded immune cell infiltration estimates for 33 tumor types from TIMER2.0 and used CIBERSORT (https://cibersort.stanford.edu/) to calculate the relative proportions of 22 infiltrating immune cells in each tumor. Spearman correlation analysis was performed to explore the relationship between PKNOX1 expression and the proportions of these immune cells, and the results are presented in a heatmap. Finally, the top five positively and negatively correlated immune cells are presented as a scatter plot. In the above analysis, a p value < 0.05 was considered statistically significant.

### Relationship between PKNOX1 expression and ICI treatment response

The results of the correlation analysis between PKNOX1 expression and tumor immune infiltration in this study revealed that PKNOX1 is closely related to tumor immunity. Immune checkpoint inhibitors(ICIs) are currently promising cancer treatment drugs. They are widely used to treat multiple tumor types and significantly prolong the survival of patients. Previous studies have shown that immune checkpoint-related genes are related to the efficacy of ICIs, and the tumor mutation burden(TMB) and microsatellite instability(MSI) can be used as predictive biomarkers for ICI treatment response (19, 20). Therefore, we used the Spearman’s correlation coefficient to evaluate the associations between PKNOX1 expression and 60 immune checkpoint-related genes (inhibitory genes and stimulatory genes) and visualized them via a heatmap. We also obtained TMB data and MSI data from the exome sequencing data of TCGA via “varscan2.”The correlation between TMB/MSI and PKNOX1 expression was analysed via the Spearman test and visualized as a lollipop plot. A p value < 0.05 was considered statistically significant.

### PKNOX1-related gene enrichment analysis

We first used the STRING website to obtain PKNOX1 binding proteins and performed functional enrichment analysis of the protein-protein interaction network. The gene list of PKNOX1 and 20 indicators were subsequently entered into the “Gene_Corr” module on the TIMER2.0, website to study the correlation between PKNOX1 and the list of 20 PKNOX1 binding indicators in various cancer types. The Jvenn website was used for cross-analysis of genes that bind to PKNOX1 and genes that interact with it were compared, and a Venn diagram was generated. Finally, we performed Kyoto Encyclopedia of Genes and Genomes(KEGG) enrichment analysis on PKNOX1 via R language to study its related functions in tumors.

### Cell culture

Five human hepatoma cell lines (HCCLM3, HepG2, Huh7, PLC/PRF/5, and MHCC97-H) and a normal liver cell line (THLE2) were purchased from the Cell Bank of the Chinese Academy of Sciences (Shanghai, China). Five human breast cancer cell lines(MCF-7, MDA-MB-453, MDA-MB-231, SK-BR-3, BT-474) and one normal breast cell line(MCF-10A) were also from the China National Collection of Cell Cultures. All the cells were cultured in high-glucose Dulbecco’s modified Eagle’s medium (DMEM) supplemented with 10% fetal bovine serum (FBS, ExcellBio), 100 μg/ml streptomycin, and 100 U/ml penicillin sodium (Solarbio, Beijing, China). The cells were cultured in a 5% CO2/95% air incubator at 37°C. The protein expression levels of PKNOX1 in each cell line was subsequently detected via Western blotting. The THLE2 and MCF-10A cell lines was used as a control.

### Tissue sample collection

We collected tumor tissue samples from 42 paired HCC tissues and adjacent non-tumor tissues from HCC patients who underwent tumor resection at the Second Affiliated Hospital of Nanchang University, China, between January 2022 and December 2023. The tissues were stored at -80°C until further analysis. This study was conducted in accordance with the Declaration of Helsinki and was approved by the Ethics Committee of the Second Affiliated Hospital of Nanchang University.

### Cell transfection

We designed three different small interfering RNA (siRNA) sequences to silence PKNOX1 in HCC cell lines. PKNOX1-targeting siRNAs (si-PKNOX1#1, si-PKNOX1#2, and si-PKNOX1#3) and control siRNA (si-NC) were synthesized by General Biotech (Anhui, China). siRNA transfection was performed via Lipofectamine 3000 transfection reagent (Invitrogen, ThermoFisher Scientific). After 48h of transfection, the knockdown efficiency was measured via Western blotting. Transfected HCC cells were collected for subsequent functional experiments. The siRNA sequences are listed in the supplementary material (**Supplementary Table S1**).

### Western blot analysis

We extracted total protein from HCC cells and tissues via radio immunoprecipitation assay (RIPA) lysis buffer (P0013B, Beyotime, Shanghai, China). The protein concentrations were determined via a BCA detection kit (Beyotime, Shanghai, China). Subsequently, equal amounts of protein were separated via 10% sodium dodecyl sulfate-polyacrylamide gel electrophoresis (SDS-PAGE) at a constant voltage (180V) and then transferred to a PVDF membrane at a current of 260mA.The membrane was subsequently blocked with 5% skim milk for 2h and incubated with a rabbit anti-human PKNOX1 antibody (1:1000, 10614-1-AP, Proteintech). GAPDH (1:5000, 60004-1-Ig, Proteintech) was used as an internal reference. The membrane was incubated with the primary antibody at 4°C overnight and then with the horseradish peroxidase (HRP)-labelled secondary antibody (1:10000, 550108, 550118, Zenbio, Chengdu, China) at room temperature for 1h. The membrane was washed three times with 1×TBST buffer for 15 min each time and developed via the ultrasensitive ECL chemiluminescence method (UElandy, Suzhou, China). The relative abundance of proteins was quantitatively evaluated via grayscale scanning via ImageLab analysis software (version 4.0, Bio-Rad) and ImageJ.

### Cell proliferation assay

Cell Counting Kit-8 (CCK8) detection: Equal amounts of treated and untreated cells (density 3×10^3^cells/well) were seeded into 96-well plates for culture. After the cells had adhered,10μl of CCK8 (UElandy, Suzhou, China) solution was added at 0, 24, 48, 72h, respectively, and the cells were transferred to a cell culture incubator and cultured for 2h. At the end of the culture period, the 96-well plates were removed, the absorbance values ​​were measured via a microplate reader set at 450nm to calculate the cell activity, and growth curves were drawn on the basis of these values.

EdU detection: Equal amounts of treated and untreated cells (density 5×10^4^cells/well) were seeded in 96-well plates and cultured for 24h. The culture medium containing the 1×EdU working mixture was then replaced, and the cells were cultured for another 2h. The cells were then fixed with 4% paraformaldehyde and washed with PBS. Finally, the cells were treated with PBS permeabilization solution containing TritonX-100. Click reaction mixture (EpiZyme, Shanghai, China) was added, and the mixture was incubated at room temperature in the dark for 30min. Then, Hoechst33342 solution was then added, and the samples were incubated at room temperature in the dark for 15min. After incubation, the cells were washed with PBS. Finally, the cells were observed and photographed under a fluorescence microscope, and the percentage of EdU positive cells was calculated as the ratio of EdU fluorescent cells to total cells in the same field of view.

### Wound healing and transwell assays

Wound healing assay: First, treated and untreated cells were evenly seeded in 6-well plates and cultured until 100% confluence was reached. The cell monolayer was then gently scratched with a sterile 200μl pipette tip and washed with PBS to remove floating cells. Fresh culture medium was then added, and the cells were cultured for 48h. At 0 and 48h after cell scratching, cell migration at the same location was observed under a microscope.

Transwell assay: Transwell chambers precoated with Matrigel (YB356234, BD Biosciences, USA) were used for invasion experiments, and chambers not coated with Matrigel were used for migration experiments. Before the cells were collected, the Matrigel gel was diluted with serum-free medium at a ratio of 1:8. Next, 60μl of diluted Matrigel was added to the upper chamber, which was placed in a 24-well plate and incubated at 37°C for 2-3h until solidification. Next, 200μl of cells (2×10^4^cells) in serum-free DMEM was added to the upper chamber, and 600μl of DMEM containing 10% FBS was added to the lower chamber. After 48h of incubation at 37°C, the luminal cells were fixed with 4% formaldehyde at room temperature for 30min and stained with crystal violet at room temperature for 15min. The cells on the upper surface of the cavity (nonmigrated cells) were wiped off with a wet cotton swab. Cell migration or invasion was recorded by measuring and photographing the cells under an inverted microscope.

### Colony formation assay

Cells (500 cells each) were seeded into 6-well plates and cultured in an incubator. After seven days of inoculation, the old culture medium was replaced, and the cells were cultured for another seven days. After culturing, the cells were gently washed twice with PBS, fixed with 4% paraformaldehyde for 30min, and stained with crystal violet for 30min. After staining, the cells were washed twice with ddH_2_O, and the remaining water was dried at room temperature. Finally, the cells were photographed and the number of cell colonies was counted.

### Statistical analysis

Bioinformatics analysis was performed via R 4.3.0. The Wilcoxon rank sum test was used to compare gene expression differences between the two groups. Survival analysis was performed via the Kaplan–Meier method and Cox regression analysis. Spearman or Pearson correlation analysis was used to elucidate inter-group correlations. Data analysis was performed via GraphPad Prism9.5 software. Differences between the two groups were compared via an unpaired t-test. P values ​​less than 0.05 were considered statistically significant.

## Results

### Expression levels of PKNOX1 in human pan-cancer, normal tissues and cell lines

**Figure 1:**
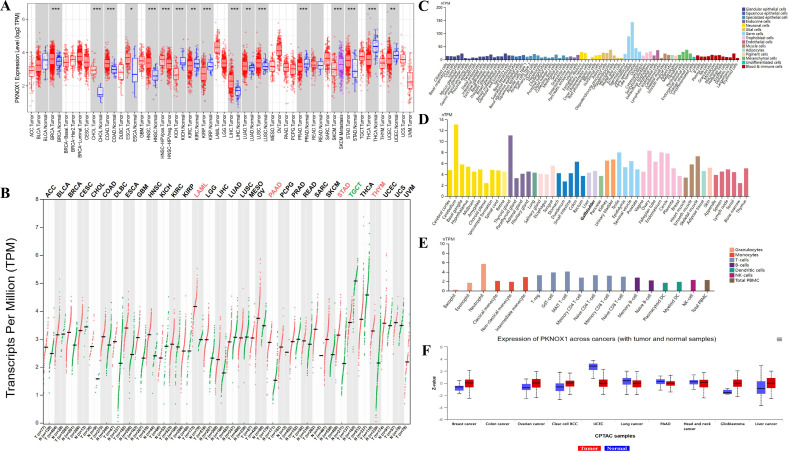
Differential expression of PKNOX1. **(A)** Expression of PKNOX1 in pan-cancer tissues in the TCGA database **(B)** Expression of PKNOX1 in pan-cancer tissues in the TCGA and GTEx databases **(C)** Expression of PKNOX1 mRNA in different single cell types in the HPA database **(D)** Expression of PKNOX1 mRNA in different human tissues in the HPA database **(E)** Expression of PKNOX1 mRNA in different blood cell lineages in the HPA database **(F)** Expression of PKNOX1 protein in different tumors and adjacent normal tissues in the CPTAC database.

We evaluated the expression of PKNOX1 mRNA in tumors and adjacent normal tissues. To explore the difference in the expression of PKNOX1 between tumors and adjacent normal tissues, we first analysed the expression level of PKNOX1 mRNA in all tumors in the TCGA database via TIMER2.0. The results showed that PKNOX1 was highly expressed in BRCA(breast invasive carcinoma), CHOL(cholangiocarcinoma)), COAD(colon adenocarcinoma), ESCA(esophageal carcinoma), HNSC(head and neck squamous cell carcinoma), LIHC(liver hepatocellular carcinoma), LUAD(lung adenocarcinoma), LUSC((lung squamous cell) and STAD((stomach adenocarcinoma) (**Figure 1A**). Since some tumor types in the TCGA database lack normal tissue or have a small number of normal tissue samples, we further analysed them via GEPIA2, which includes both the TCGA and GTEx databases, and expanded the sample size. Compared with adjacent normal tissues, in addition to the above 9 tumor types expressing higher levels of PKNOX1, PKNOX1 was also highly expressed in ACC(adrenocortical cancer), DLBC(diffuse large B-cell lymphoma), OV(ovarian serous cystadenocarcinoma), PAAD(pancreatic adenocarcinoma), UCEC (uterine corpus endometrioid carcinoma), GBM (glioblastoma multiforme),PCPG (pheochromocytoma&paraganglioma),UCS (uterine carcinosarcoma), LAML (acute myeloid leukemia), LGG (brain lower grade glioma), SARC (sarcoma), SKCM (skin cutaneous melanoma), and THYM (thymoma) (**Figure 1B**). These data show that PKNOX1 is highly expressed in the vast majority of tumors.

We used the HPA website to analyse the expression level of PKNOX1 mRNA in different human tissues and cell lines. The results revealed that PKNOX1 is widely expressed in different human tissues, single-cell types and blood cell lineages, and has low expression specificity in different human tissues and cell lines. PKNOX1 was relatively highly expressed in germ cells, cerebellar, thyroid and immune cells, and was expressed at relatively low levels in cones, bone marrow and basophils (**Figures 1C–E**).

We used the CPTAC database from the UALCAN website to analyse the protein expression levels of PKNOX1 in tumors and adjacent normal tissues. The results revealed that the total protein expression level of PKNOX1 in primary tumor tissues from patients with breast cancer, ovarian cancer, clear cell carcinoma, glioblastoma, and liver cancer was greater than that in adjacent normal tissues (**Figure 1F**).

### Expression and subcellular localization of PKNOX1 in tumors

**Figure 2:**
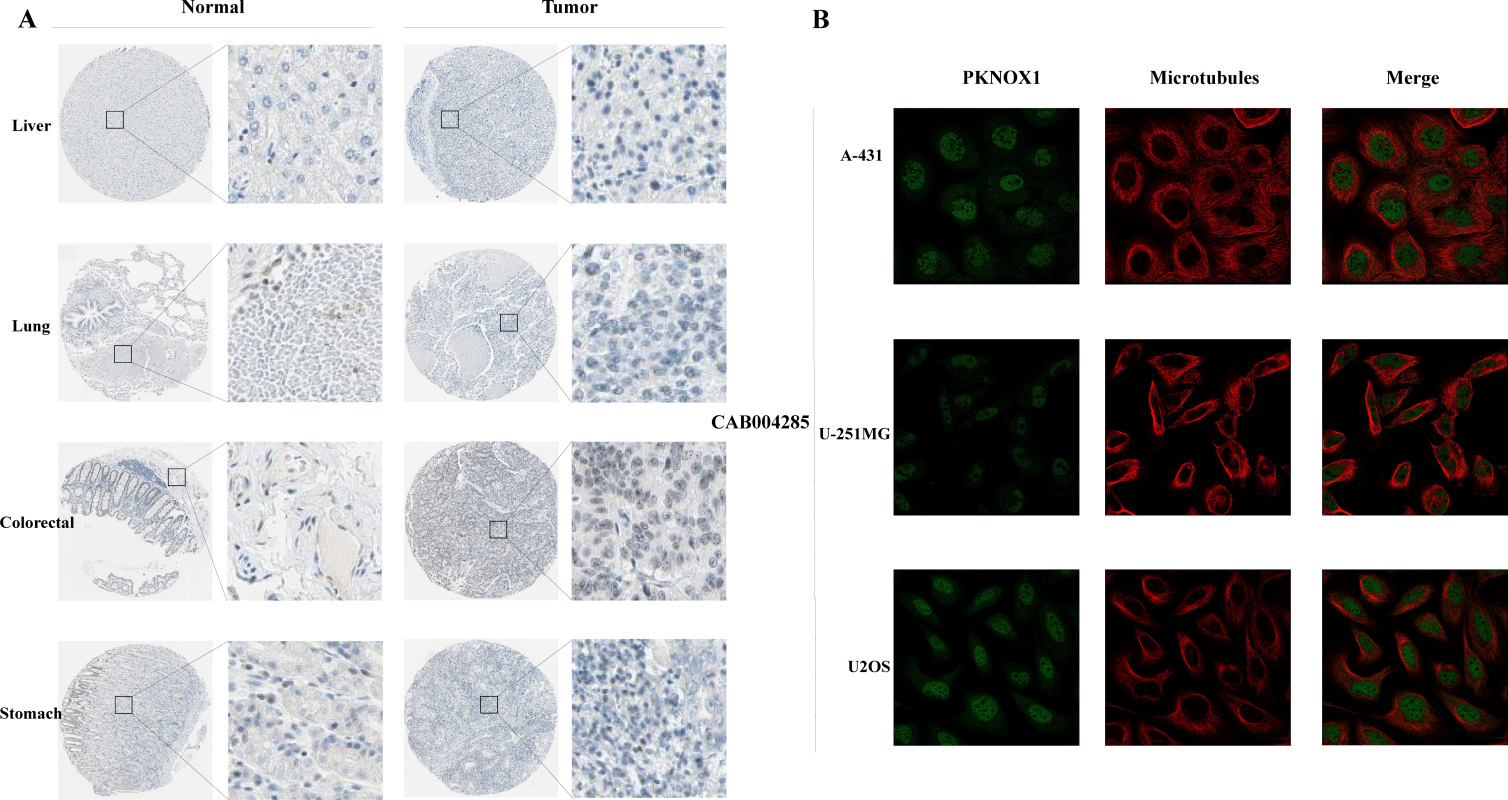
Expression and subcellular localization of PKNOX1 in tumors. **(A)** Immunohistochemical staining of PKNOX1 protein expression in liver cancer, lung cancer, colorectal cancer, and gastric cancer tissues from the HPA database. **(B)** Immunofluorescence staining of PKNOX1 subcellular localization was analysed in A-431, U-251MG and U2OS cells from the HPA database.

We used the HPA website to further analyse the differential expression of PKNOX1 in tumor tissues and adjacent normal tissues through immunohistochemistry. Compared with that in adjacent normal tissues, PKNOX1 expression was significantly increased in LIHC, lung cancer, colorectal cancer, and gastric cancer tissues (**Figure 2A**). We also found that PKNOX1 was localized to the nuclei of A-431, U-251MG, and U2OS cells (**Figure 2B**).

### Gene mutation characteristics and DNA modifications of PKNOX1 in pan-cancer

**Figure 3:**
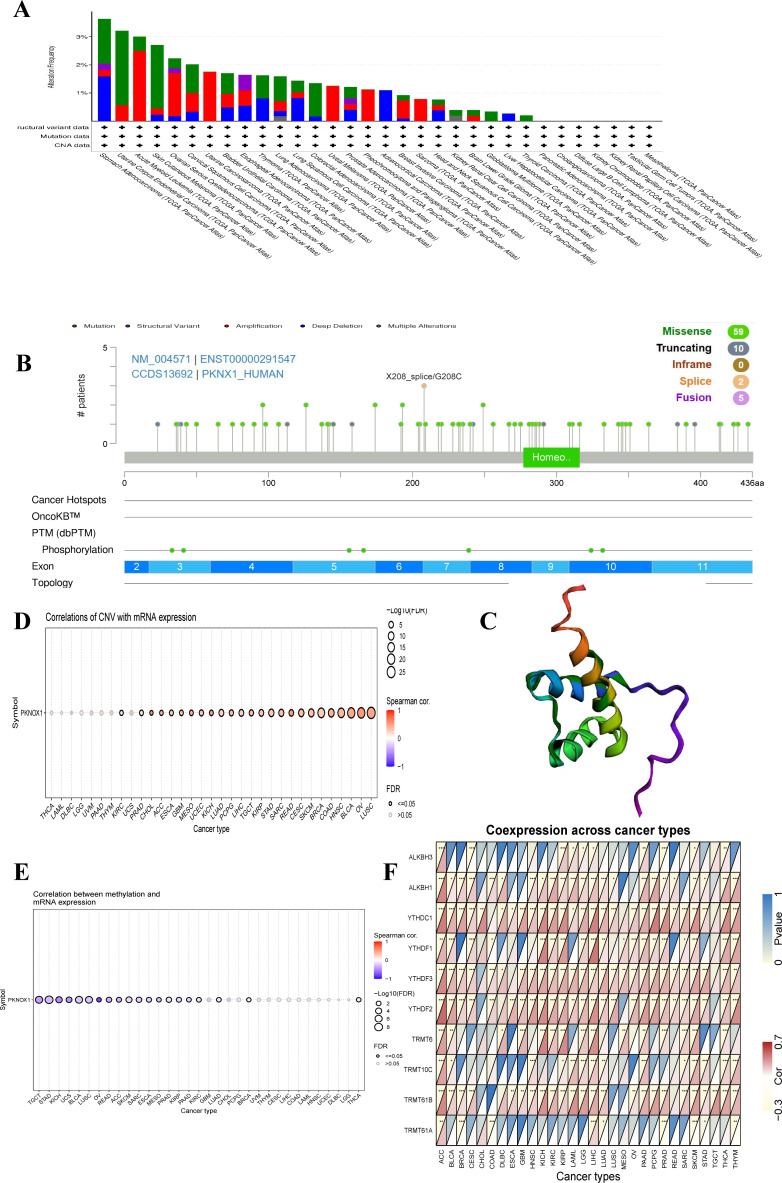
Mutation characteristics of PKNOX1 in pan-cancer. **(A)** Mutation types and mutation frequencies of PKNOX1 in different tumors. **(B, C)** Somatic mutation frequency and post-translational modification sites of PKNOX1 and 3D structure of PKNOX1. **(D, E)** Correlations of PKNOX1 expression level with CNV and DNA methylation in pan-cancer. **(F)** Correlations of PKNOX1 expression with 9 methylation-related genes in pan-cancer. CNV, copy number variation.

To investigate the genetic variation in PKNOX1 in pan-cancer, we used the cBioPortal and GSCALite online analysis webpages to analyse the genetic variation frequency, mutation type, and degree of DNA methylation. The results revealed that the highest frequency of PKNOX1 gene mutation was in gastric adenocarcinoma (3.64%), of which 7 cases (1.59%) had “mutation” and 7 cases (1.59%) had “deep deletion.” “Mutation” was most frequently found in uterine corpus endometrial carcinoma (2.65%), whereas “structural variant,” “amplification,” “deep deletion” and “multiple alterations” were commonly found in esophageal adenocarcinoma (0.55%), acute myeloid leukemia (2.5%), gastric adenocarcinoma (1.59%), and kidney renal clear cell carcinoma (0.2%), respectively (**Figure 3A**). The types, locations, number of cases, and phosphorylation sites of the PKNOX1 gene mutations are shown in **Figure 3B**, and the 3D model diagram is shown in **Figure 3C**. We found that the frequency of somatic mutations in PKNOX1 was 0.6%, and missense mutation, the main type of gene mutation, were detected in 59 patients (77.6%). In addition, truncated mutations were detected in 10 patients (13.2%), splicing mutations were detected in 2 patients (2.6%), and fusions were detected in 5 patients (6.6%) (**Figure 3B**).

The correlations between PKNOX1 expression levels and CNVs and DNA methylation were analysed via the Pearson test on the GSCALite platform. The results revealed that PKNOX1 expression levels were significantly positively correlated with the percentage of CNVs in 25 tumor types (**Figure 3D**). Current studies have shown that DNA methylation can cause changes in chromatin structure, DNA conformation, DNA stability, and how DNA interacts with proteins, thereby inhibiting gene expression (21, 22). Correlation analysis revealed that PKNOX1 expression levels were significantly negatively correlated with methylation levels in 19 tumor types (**Figure 3E**), indicating that low DNA methylation may be one of the reasons for the high pan-cancer expression of PKNOX1. We further analysed the relationships between PKNOX1 expression and the expression of nine methylation-related genes (ALKBH3, ALKBH1, YTHDC1, YTHDF1, YTHDF3, YTHDF2, TRMT6, TRMT10C, TRMT61B, TRMT61A). The results showed that PKNOX1 expression was more closely related to YTHDC1 and YTHDF3 expression. There was a significant positive correlation between PKNOX1 expression and YTHDC1 and YTHDF3 expression in 27 tumor types. No tumor types with significant negative correlations were observed. PKNOX1 expression has the weakest correlation with TRMT61A. PKNOX1 expression was significantly positively correlated with nine methylation-related genes in ACC and LIHC, significantly positively correlated with eight methylation-related genes in SKCM, and significantly negatively correlated with one gene (TRMT61A), only one gene (YTHDC1) was significantly positively correlated with CHOL (**Figure 3F**).

### Correlation analysis between PKNOX1 and clinicopathological features of various tumor types

**Figure 4:**
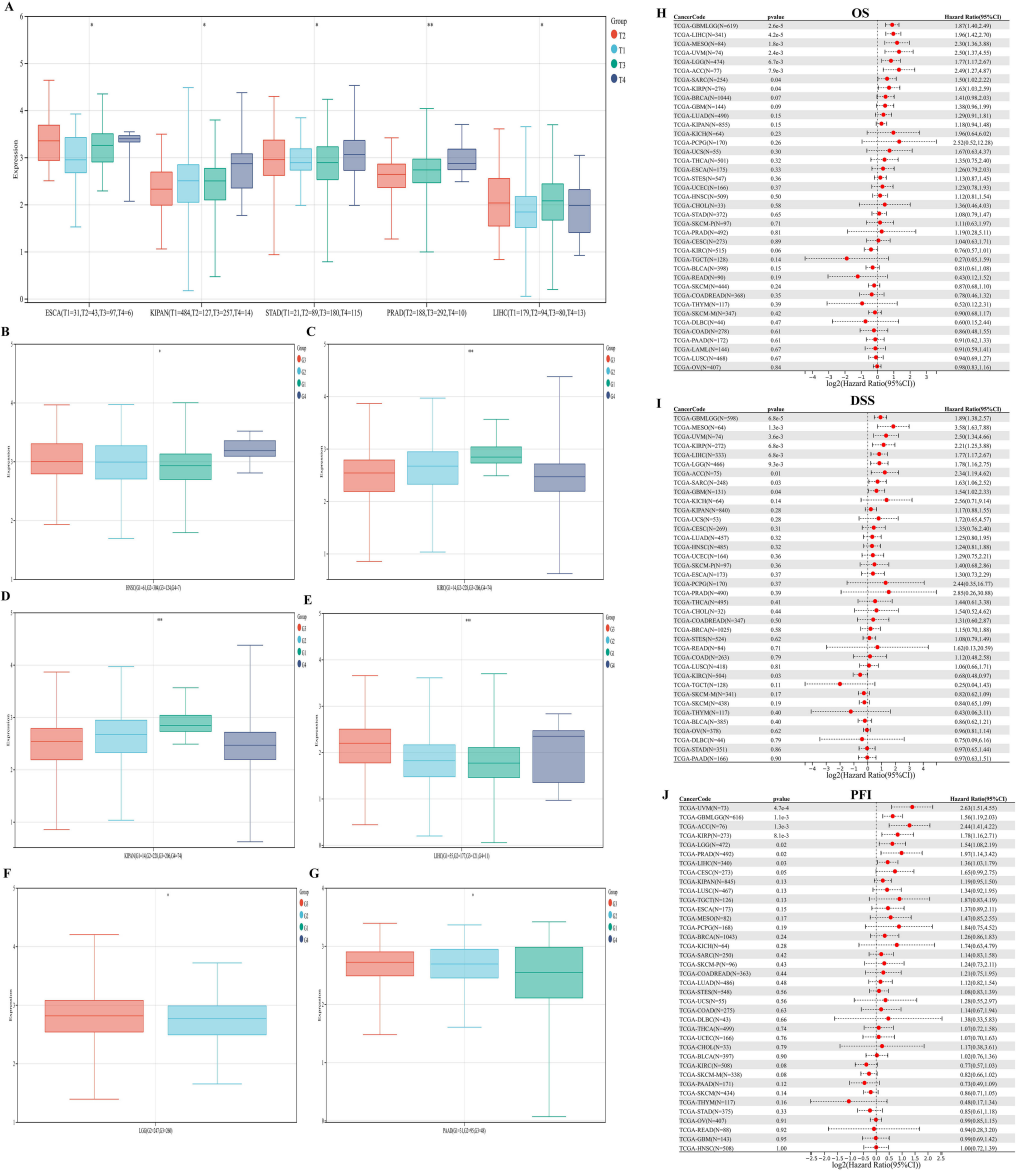
Correlations between PKNOX1 expression and clinical pathological characteristics of different tumors and Cox regression analysis of survival prognosis. **(A)** Correlations between PKNOX1 expression and clinical T stage of ESCA, KIPAN, STAD, PRAD and LIHC tumors. **(B–G)** Correlations between PKNOX1 expression and the pathological G stage of HNSC, KIRC, KIPAN, LIHC, LGG and PAAD tumors. **(H–J)** Relationships between PKNOX1 expression and pan-cancer OS, DSS and PFI according to univariate Cox regression analysis.

We evaluated the associations between PKNOX1 expression and clinicopathological features in different tumor types and compared the correlations between clinical T stage and pathological G stage (which assesses the grade of tumor malignancy) and PKNOX1 expression levels. The results revealed that patients with advanced clinical T stage (T3&T4) showed higher levels of PKNOX1 expression in ESCA, KIPAN(Pan-kidney cohort), STAD, PRAD and LIHC tumors (**Figure 4A**), and patients with advanced pathological G stage (G3/G4) showed higher levels of PKNOX1 expression in HNSC, KIRC, KIPAN, LIHC, LGG, and PAAD tumors (**Figures 4B–G**). In conclusion, PKNOX1 may promote tumor progression and lead to poor prognosis in LIHC.

### Prognostic value of PKNOX1 in pan-cancer

**Figure 5:**
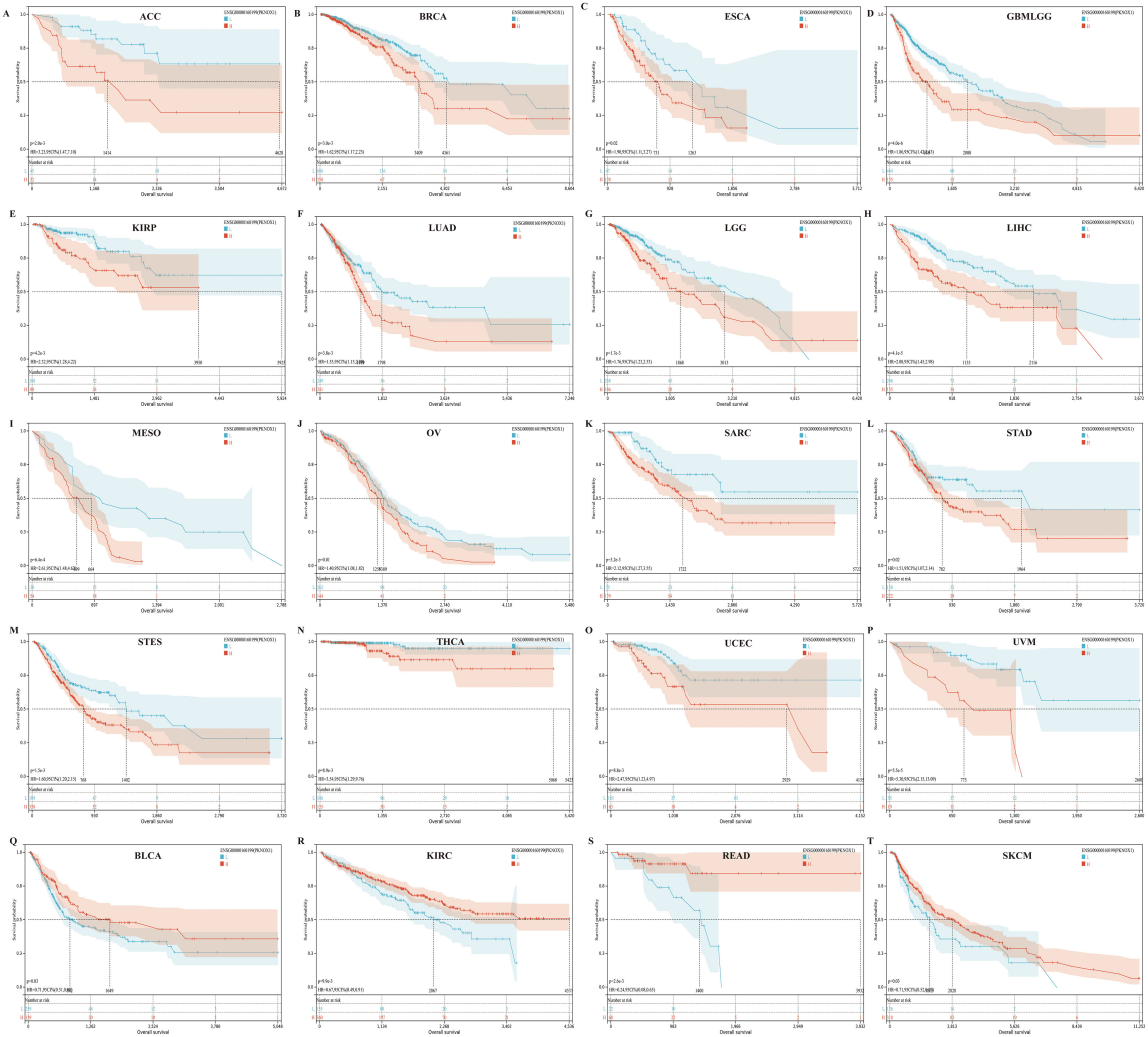
There is a significant association between PKNOX1 expression and the overall survival rate of 20 tumors according to Kaplan-Meier analysis. **(A)** ACC **(B)** BRCA **(C)** ESCA **(D)** GBMLGG **(E)** KIRP **(F)** LUAD **(G)** LGG **(H)** LIHC **(I)** MESO **(J)** OV **(K)** SARC **(L)** STAD **(M)** STES **(N)** THCA **(O)** UCEC **(P)** UVM **(Q)** BLCA **(R)** KIRC **(S)** READ **(T)** SKCM.

We divided patients into high expression groups and low expression groups according to the expression level of PKNOX1, and evaluated the correlation between PKNOX1 expression and the prognosis of patients with different tumors via Kaplan-Meier survival analysis and univariate Cox regression analysis. Univariate Cox regression analysis revealed that high expression of PKNOX1 was a prognostic risk factor for OS in patients with GBMLGG(Glioma), LIHC, MESO, UVM, LGG, ACC, SARC, and KIRP (**Figure 4H**); it was a prognostic risk factor for DSS in patients with GBMLGG, LIHC, MESO, UVM, LGG, ACC, SARC, KIRP, and GBM (**Figure 4I**); and it was a prognostic risk factor for PFI in patients with GBMLGG, LIHC, UVM, LGG, ACC, PRAD, and KIRP (**Figure 4J**). K-M analysis results revealed that high PKNOX1 expression was associated with worse OS (**Figures 5A–P**), DSS (**Supplementary Figure S1**) and PFI (**Supplementary Figure S2**) in patients with ACC, GBMLGG, KIRP, LGG, LIHC, MESO, SARC, UVM, and OV tumors, which was consistent with the results of Cox regression analysis. In contrast, in BLCA, KIRC, READ and SKCM (**Figures 5Q–T**), low PKNOX1 expression was significantly associated with poor OS, and in KIRC, low PKNOX1 expression was significantly associated with poor OS, DSS and PFI overall (**Figure 5R**; **Supplementary Figure 1Q**, **Supplementary Figure 2R**). Overall, high PKNOX1 expression is a prognostic risk factor for OS (P=4.2e-5), DSS (P=6.8e-3), and PFI (P=0.03) in LIHC patients, and is associated with poor OS (P=4.1e-5), DSS (P=1.4e-4), and PFI (P=5.0e-4), indicating that PKNOX1 is closely related to the poor prognosis of LIHC patients and can be used as a prognostic indicator. Moreover, high expression of PKNOX1 is closely associated with poor prognosis in other pan-cancers, suggesting that PKNOX1 is a promising pan-cancer prognostic biomarker.

### Correlation analysis of PKNOX1 expression with pan-cancer TME and immune infiltration levels

**Figure 6:**
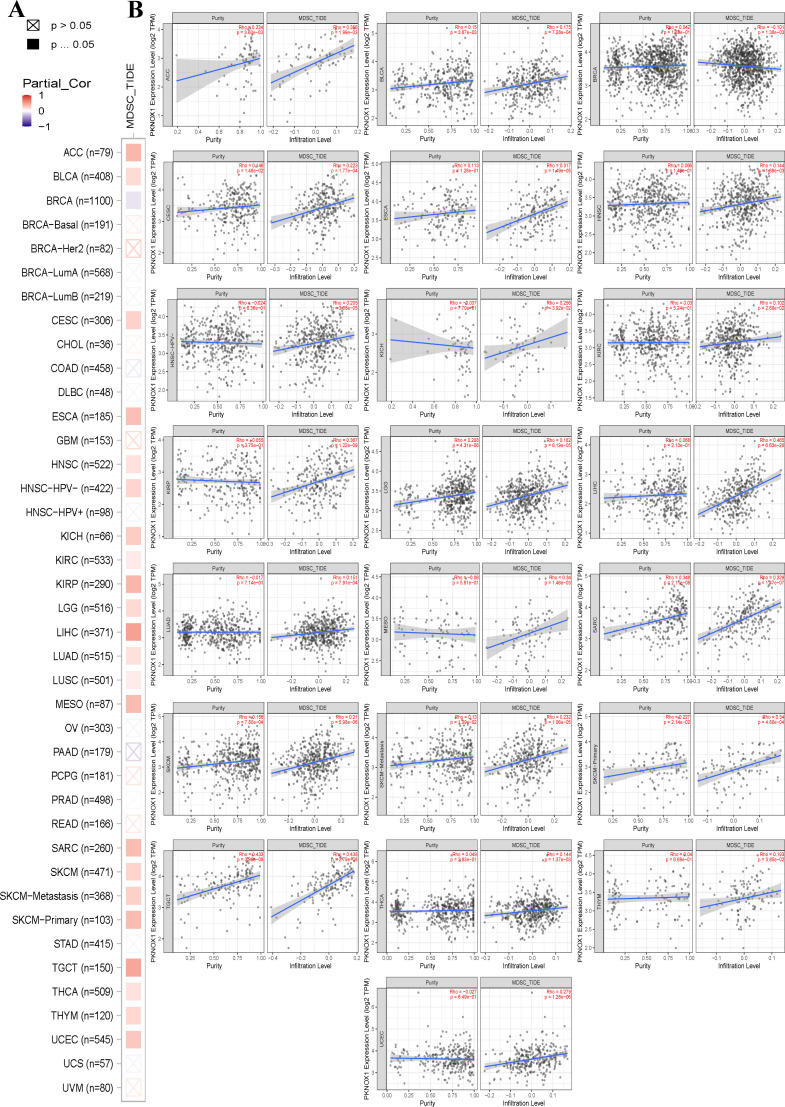
Correlation analysis between PKNOX1 expression and MDSC immune infiltration. **(A)** Relationships between MDSC infiltration levels and PKNOX1 gene expression in all tumor types. **(B)** Scatter plot showing the correlation between the MDSC infiltration level and PKNOX1 gene expression.

The tumor immune microenvironment (TME) is closely related to the occurrence and development of tumors. Immune cells and stromal cells are important components of the TME. Our previous analysis revealed that PKNOX1 is highly expressed in immune cells (23). It has been reported that myeloid-derived suppressor cells (MDSCs) can promote immunosuppressive effects by inhibiting effector T cells and natural killer (NK) cells and stimulating other suppressive immune cells, thereby achieving tumor immune escape and promoting tumor progression and metastasis (24, 25). Therefore, we investigated the correlation between PKNOX1 expression levels and the infiltration of various immune cell subsets in the TME. We found that in most tumor types (22 types), PKNOX1 expression levels were positively correlated with the infiltration levels of MDSCs (**Figures 6A, B**).

**Figure 7:**
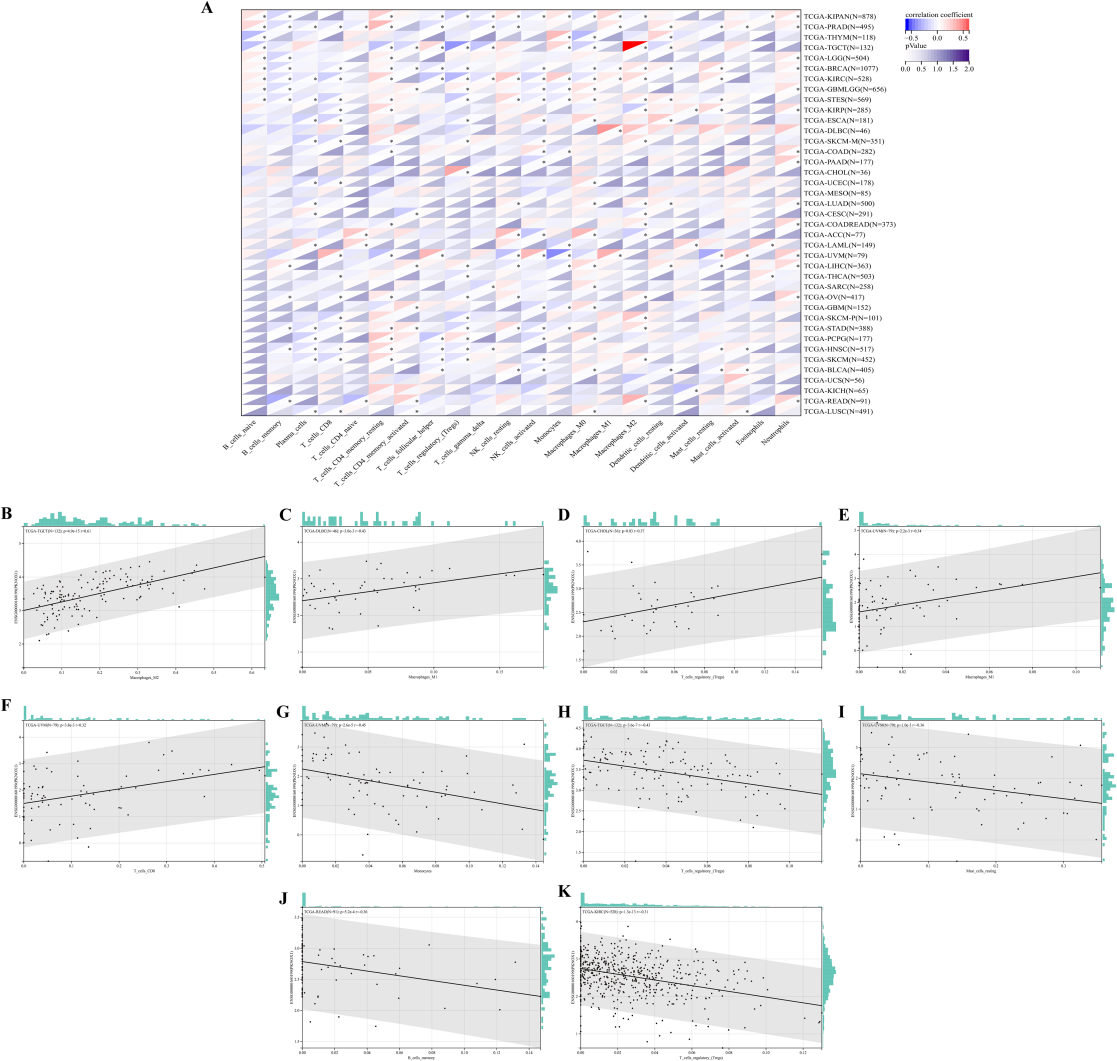
Correlation analysis of PKNOX1 expression and immune infiltrating cells in pan-cancer based on the CIBERSORT algorithm. **(A)** Correlation analysis between PKNOX1 expression and the proportions of 22 types of infiltrating immune cells. **(B–F)** The top five tumors with the most significant positive correlations between PKNOX1 expression and immune cells. **(G–K)** The top five tumors showing the most significant negative correlations between PKNOX1 expression and immune cells.

Next, we explored the correlation between immune cell type and PKNOX1 expression levels. The results revealed that PKNOX1 expression was most strongly correlated with the level of immune infiltration in KIRC. Correlation analysis revealed that PKNOX1 was positively correlated with naive B cells, resting memory CD4 T cells, resting NK cells, M1 and M2 macrophages, resting dendritic cells, activated mast cells, and neutrophils, and negatively correlated with memory B cells, plasma cells, CD8 T cells, follicular helper T cells, regulatory T cells, γδT cells, and activated NK cells. In most tumors, resting memory CD4 T cells, resting NK cells, M2 macrophages, and neutrophils positively correlated with PKNOX1, whereas plasma cells, CD8 T cells, regulatory T cells, and activated NK cells negatively correlated with PKNOX1 (**Figure 7A**), suggesting that PKNOX1 may be associated with tumor immunosuppression. We constructed the scatter plots of the top five tumor types with positive and negative correlations between immune cells and PKNOX1 expression. The results revealed that PKNOX1 was negatively correlated with monocytes in UVM, resting mast cells, regulatory T cells in TCGT and KIRC, and memory B cells in READ, and positively correlated with M1 macrophages in DLBC and UVM, M2 macrophages in TGCT, regulatory T cells in CHOL, and CD8 T cells in UVM (**Figures 7B–K**).

### Analysis of the ability of PKNOX1 to predict ICIs treatment response

**Figure 8:**
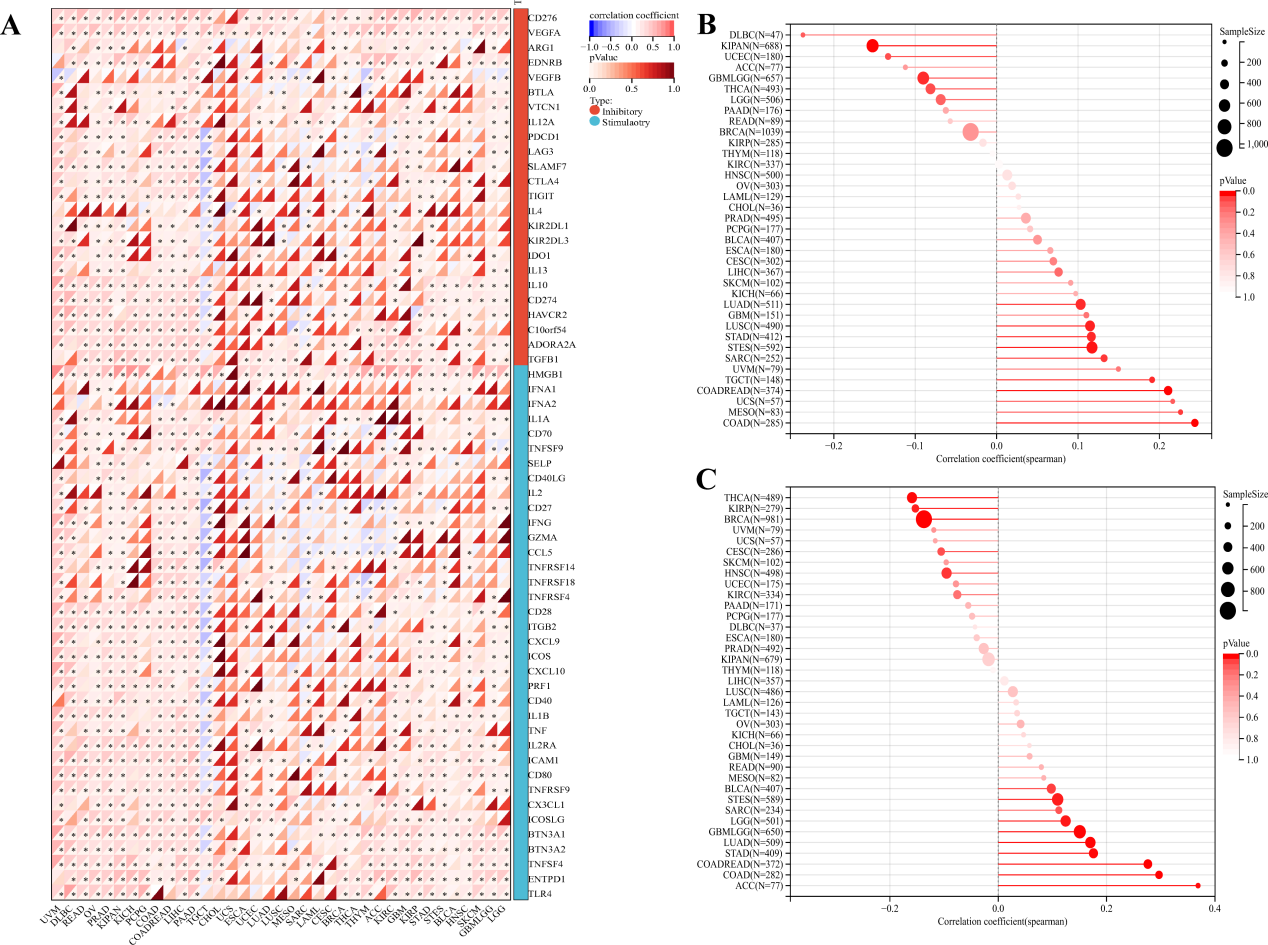
Relationships between PKNOX1 expression and pan-cancer immune checkpoint inhibitor treatment. **(A)** Relationships between PKNOX1 expression and the expression of 60 immune checkpoint-related genes. **(B)** Relationships between PKNOX1 expression and tumor mutation burden (TMB). **(C)** Correlation between PKNOX1 expression and microsatellite instability (MSI).

The above analysis revealed that PKNOX1 may be related to tumor immunity. In recent years, immune checkpoint inhibitors (ICIs) have played an important role in tumor treatment as a promising emerging treatment methods, and immune checkpoint-related genes can be used as effective predictors of ICIs (5, 26). We extracted the expression data of 37 tumor types from the TCGA database and analysed the relationships between PKNOX1 expression and 60 immune checkpoint-related genes. The results revealed that PKNOX1 was significantly associated with immune checkpoint-related genes in most tumors. In LIHC, in addition to ARG1, EDNRB, KIRZDL1, IFNA1, IFNA2, SELP, and PRF1, PKNOX1 was positively correlated with 53 of the 60 genes, whereas fewer genes were correlated between with PKNOX1 and immune checkpoint-related genes in UCS and CHOL samples (**Figure 8A**). TMB and MSI are closely associated with the therapeutic effects of immune checkpoint inhibitors. We further explored the correlation among PKNOX1 expression and TMB and MSI. The results revealed that in GBMLGG, LGG, LUAD, COAD, COADREAD(Colon adenocarcinoma/Rectum adenocarcinoma Esophageal carcinoma), STES, STAD, BLCA, and ACC, the expression level of PKNOX1 was significantly positively correlated with TMB. According to the Spearman correlation coefficient, the correlation with TMB was strongest for ACC (R=0.37, P=9.6e-4). There was a significant negative correlation in BRCA, KIRP, HNSC, and THCA (**Figure 8B**). In MESO, TGCT(testicular germ cell tumors), LUAD, COAD, COADREAD, STES, STAD, SARC, and LUSC, the expression level of PKNOX1 was significantly positively correlated with MSI, whereas it was significantly negatively correlated with GBMLGG and KIPAN (**Figure 8C**). These results suggest that PKNOX1 can serve as a predictor of the response to ICIs treatment in certain tumors.

### PKNOX1 related gene enrichment analysis

**Figure 9:**
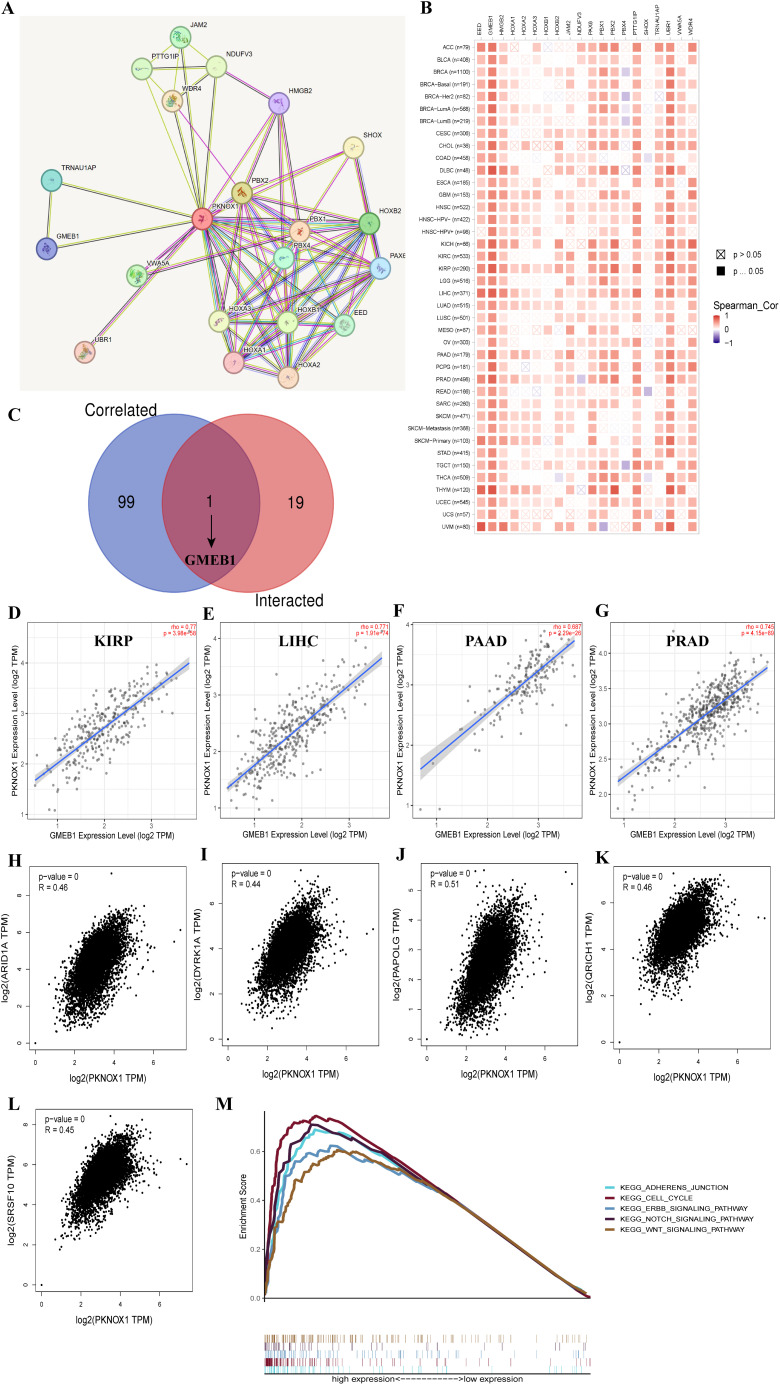
Enrichment analysis of PKNOX1-related genes. **(A)** Prediction analysis of PKNOX1-interacting proteins. **(B)** Correlation analysis of PKNOX1 and 20 interacting proteins bound by PKNOX1 in pan-cancer. **(C)** Cross-analysis of PKNOX1-related genes and PKNOX1-interacting genes. **(D–G)** Correlation analysis of PKNOX1 and GMEB1. **(H–L)** Through GEPIA2, we obtained the top 100 PKNOX1-related genes in the TCGA database and analysed the expression correlation between PKNOX1 and selected target genes (ARID1A, DYRK1A, PAPOLG, QRICH1, SRSF10). **(M)** KEGG pathway enrichment analysis of PKNOX1.

We screened for targeted PKNOX1-binding proteins and PKNOX1 expression-related genes to further study the molecular mechanism of the PKNOX1 gene in tumors. Using the STRING tool, we identified 20 PKNOX1-binding proteins, including proteins supported by experimental evidence (**Figure 9A**). We further analysed the correlations between the expression of PKNOX1 and 20 PKNOX1-binding proteins, and found that EED, GMEB1, HMGB2, PAX6, PTTG1IP, UBR1, WDR4, and PKNOX1 were significantly positively correlated in most tumors (**Figure 9B**). We subsequently used GEPIA2 to merge the expression data of all the tumors in the TCGA database and obtained the top 100 genes related to PKNOX1 expression. The expression level of PKNOX1 was closely related to ARID1A (R=0.46), DYRK1A (R=0.44), PAPOLG (R=0.51), QRICH1 (R=0.46) and SRSF10 (R=0.45) which were significantly positively correlated (**Figures 9H–L**). A common member, GMEB1, was discovered via cross-analysis (**Figure 9C**), and further analysis revealed that GMEB1 was strongly positively correlated with PKNOX1 expression (KIRP, LIHC, PAAD and PRAD are listed) (**Figures 9D–G**). We used R language to perform Kyoto Encyclopedia of Genes and Genomes (KEGG) pathway enrichment analysis and found that PKNOX1 was involved mainly in the intercellular adhesion junction pathway, the cell cycle pathway, the ERBB signalling pathway, the NOTCH signalling pathway and the WNT signalling pathway (**Figure 9M**), indicating that PKNOX1 may promote tumor occurrence and development by promoting tumor proliferation, migration and invasion.

### PKNOX1 is highly expressed in HCC and promotes the proliferation, migration and invasion of HCC cells

**Figure 10:**
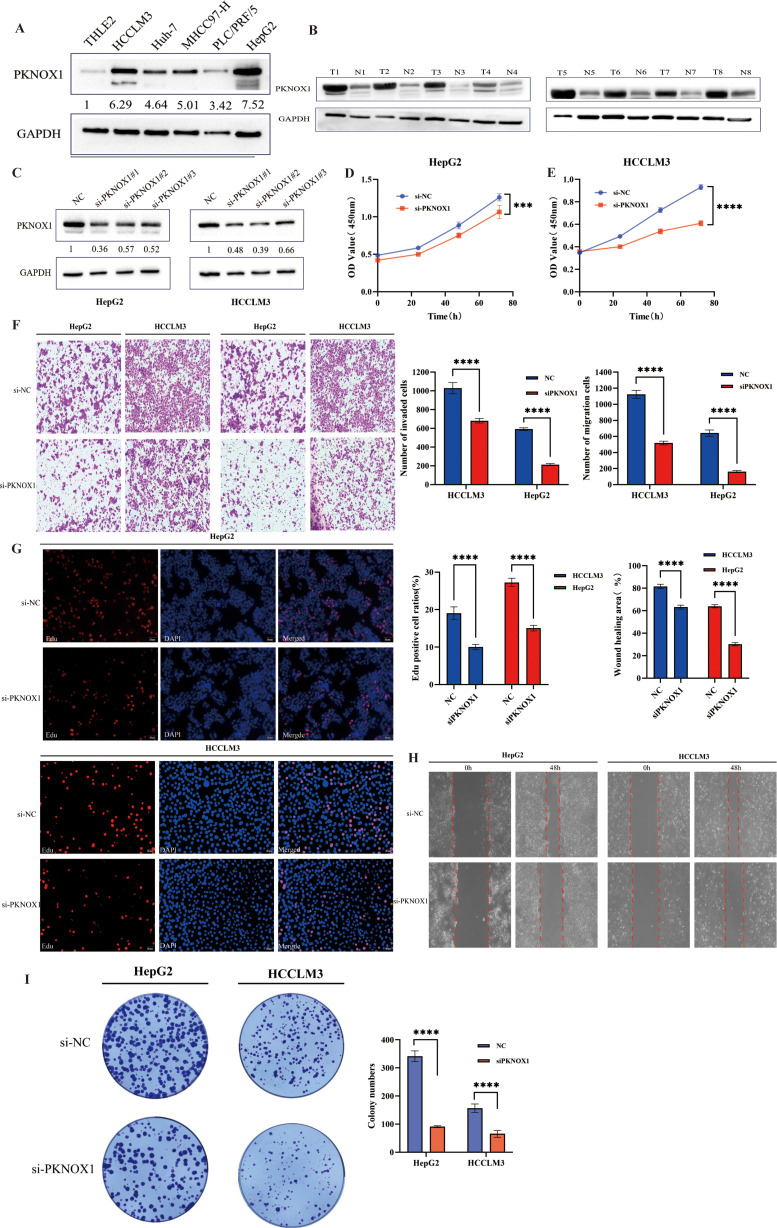
Differential expression of PKNOX1 in HCC cells and tissues and its effects on HCC cell proliferation, migration and invasion. **(A)** Western blotting analysis of PKNOX1 protein expression in five HCC cell lines and normal hepatocytes. The values ​​are expressed as the relative expression level of the PKNOX1 protein. **(B)** Eight pairs of HCC tumor tissues and corresponding adjacent normal tissues were randomly selected, and the protein expression of PKNOX1 was analysed via Western blotting. **(C)** Western blotting was used to evaluate the inhibitory efficiency of different siRNA-PKNOX1 in HepG2 and HCC-LM3 cells. **(D, E)** CCK8 assay was used to evaluate the proliferation ability of HepG2 and HCC-LM3 cells. **(F)** Transwell assay was used to evaluate the cell migration and invasion ability of HepG2 and HCC-LM3 cells. **(G)** EdU assay was used to evaluate the cell proliferation ability of HepG2 and HCC-LM3 cells. **(H)** Scratch assay was used to evaluate the migration ability of HepG2 and HCC-LM3 cells. **(I)** Clone formation assay was used to evaluate the growth and proliferation ability of HepG2 and HCC-LM3 cells. ***P<0.001, ****P<0.0001.

Our previous pan-cancer analysis revealed that PKNOX1 is highly expressed in HCC and is related to the OS, DSS, and PFI of HCC patients, and that there is a significant correlation between PKNOX1 expression and HCC tumor immunity. Considering the key regulatory role of PKNOX1 in the occurrence and development of HCC, we further explored the biological function of PKNOX1 in HCC. We first analysed the differential expression of PKNOX1 in HCC cell lines (HCC-LM3, HepG2, Huh7, PLC/PRF/5, MHCC97-H) via Western Blotting (WB). Compared with thay in the normal liver cell line THLE2, PKNOX1 was significantly upregulated the five HCC cell lines (**Figure 10A**), with relatively high expression in HCC-LM3 and HepG2 cells and relatively low expression in MHCC97-H and PLC/PRF/5 cells. We collected postoperative HCC tissue and adjacent normal tissue samples from clinical HCC patients, and randomly selected 8 pairs of specimens for WB analysis. The results revealed that the expression of PKNOX1 in HCC tissue was significantly higher than that in adjacent normal tissue (**Figure 10B**), which is consistent with the TCGA database analysis results.

KEGG enrichment analysis previously revealed that PKNOX1 is involved mainly in cell-cell adhesion junctions, the cell cycle, and the ERBB, NOTC, and WNT signalling pathways, which affect tumor proliferation, migration, and invasion. Therefore, we investigated the effects of PKNOX1 on HCC cell growth, proliferation and metastasis. Given that PKNOX1 is relatively highly expressed in HCC-LM3 and HepG2 cells, we selected these two cell lines for siRNA transfection targeting PKNOX1. The interference efficiency of three siRNA fragments (si-PKNOX1#1, si-PKNOX1#2 and si-PKNOX1#3) was verified via WB, and the best knockdown efficiency siRNA for HCC-LM3 (si-PKNOX1#2) and the best knockdown efficiency siRNA for HepG2 (si-PKNOX1#1) were determined (**Figure 10C**). These two siRNAs were used to continue the subsequent functional experiments. Next, we used CCK8, EdU staining and plate cloning experiments to explore the role of PKNOX1 in HCC cell growth and proliferation. Compared to the control, PKNOX1 knockdown significantly reduced the growth and proliferation ability of HCC cells (**Figures 10D, E, G, I**). We used wound healing and Transwell assays to verify the role of PKNOX1 in HCC cell migration and invasion. In the wound healing assay, compared with that in the control group, the migration rate of PKNOX1 knockdown cells was significantly lower 48h after scratching (**Figure 10H**). The same results were also observed in the Transwell experiment without Matrigel. Transwell invasion experiments also revealed that the invasive ability of PKNOX1 knockdown cells was significantly lower than that of the control cells (**Figure 10F**). Taken together, these findings indicate that PKNOX1 promotes the growth, proliferation, migration and invasion of HCC cells.

### PKNOX1 is highly expressed in breast cancer and promotes breast cancer cell proliferation, migration, and invasion

**Figure 11:**
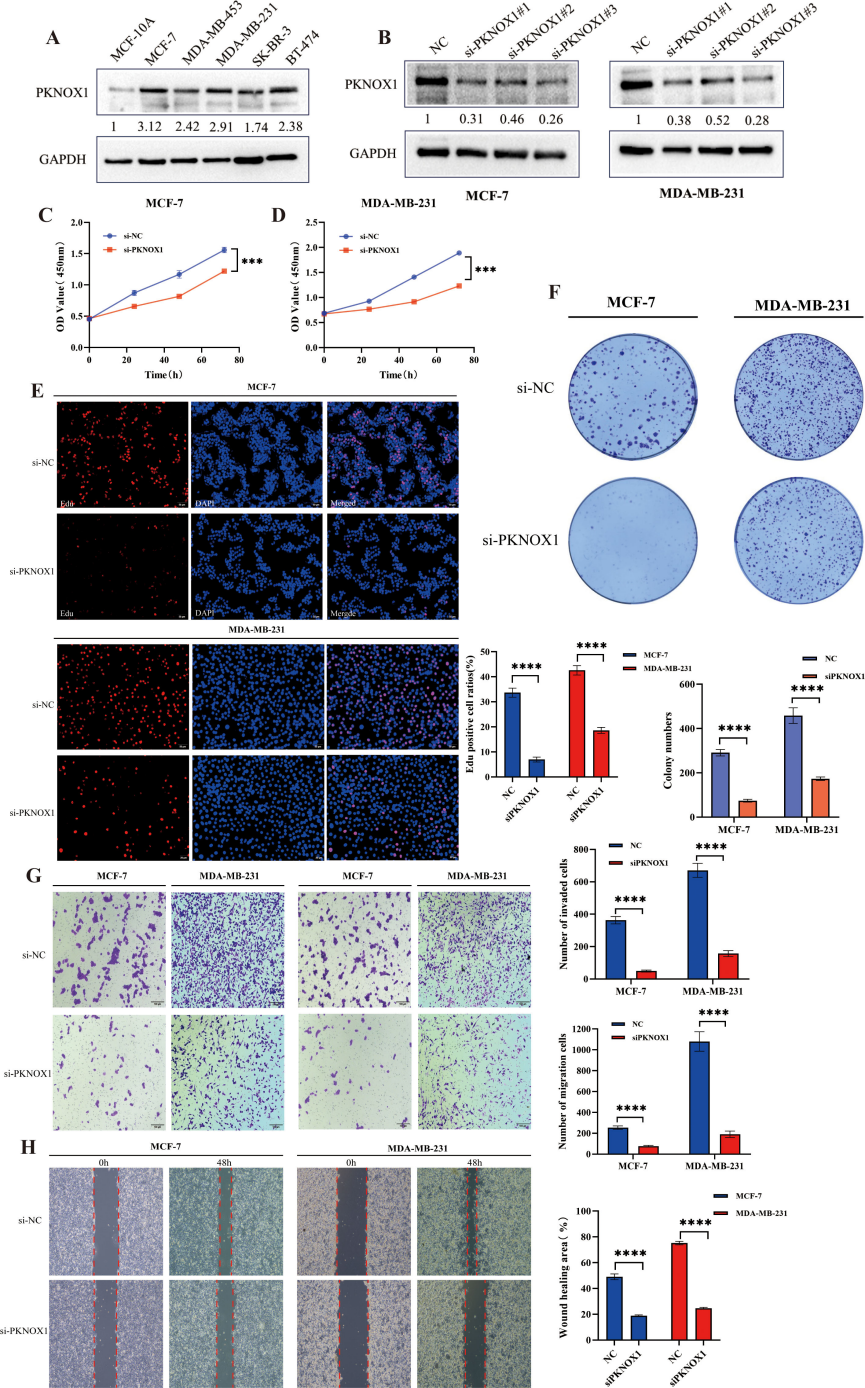
Differential expression of PKNOX1 in breast cancer cells and its effects on breast cancer cell proliferation, migration and invasion. **(A)** Western blotting analysis of PKNOX1 protein expression in five breast cancer cell lines and normal breast cell. The values ​​are expressed as the relative expression level of the PKNOX1 protein. **(B)** Western blotting was used to evaluate the inhibitory efficiency of different siRNA-PKNOX1 in MCF-7 and MDA-MB-231 cells. **(C, D)** CCK8 assay was used to evaluate the proliferation ability of MCF-7 and MDA-MB-231 cells. **(E)** EdU assay was used to evaluate the cell proliferation ability of MCF-7 and MDA-MB-231 cells. **(F)** Clone formation assay was used to evaluate the growth and proliferation ability of MCF-7 and MDA-MB-231 cells. **(G)** Transwell assay was used to evaluate the cell migration and invasion ability of MCF-7 and MDA-MB-231 cells. **(H)** wound healing assay was used to evaluate the migration ability of MCF-7 and MDA-MB-231 cells. ***P<0.001, ****P<0.0001.

We further validated the expression and function of PKNOX1 in breast cancer. WB analysis revealed that PKNOX1 was significantly upregulated in five breast cancer cell lines(MCF-7, MDA-MB-453, MDA-MB-231, SK-BR-3, BT-474) compared to the normal mammary epithelial cell line MCF-10A (**Figure 11A**). The two cell lines with the highest PKNOX1 expression, MCF-7 and MDA-MB-231, were selected for functional experiments.PKNOX1 was knocked down using siRNA transfection. WB confirmed the interference efficiency of three siRNA fragments (si-PKNOX1#1, si-PKNOX1#2, and si-PKNOX1#3), and the most effective siRNA (si-PKNOX1#3) was chosen for subsequent experiments (**Figure 11B**). CCK-8, EdU staining, and colony formation assays were performed to investigate the role of PKNOX1 in breast cancer cell proliferation. The results demonstrated that PKNOX1 knockdown significantly suppressed breast cancer cell growth and proliferation compared to the control group (**Figures 11C–F**).Wound healing and Transwell assays were conducted to examine the role of PKNOX1 in breast cancer cell migration and invasion. In the Transwell assay, PKNOX1 knockdown significantly reduced the migratory and invasive abilities of breast cancer cells (**Figure 11G**). Similarly, in the wound healing assay, the migration rate of PKNOX1-knockdown cells was markedly decreased after 48 hours (**Figure 11H**).These findings are consistent with the results observed in HCC cells, further confirming that PKNOX1 is highly expressed in most cancers and promotes tumor growth, proliferation, migration, and invasion.

## Discussion

PKNOX1, a homeodomain transcription factor of the Meniox family, is involved in growth and differentiation during embryonic development (13). Recent studies have shown that PKNOX1 dysregulation is closely related to tumor development, metastasis and poor prognosis (17, 18, 27). However, most of these studies are on a single tumor type, and there are currently no clear reports on the expression pattern, prognostic value and biological significance of PKNOX1 in most tumors. Therefore, in this study, we systematically performed a pan-cancer bioinformatics analysis of PKNOX1 via multiple databases, including gene expression analysis, gene mutation analysis, DNA methylation, prognostic value and clinical characteristic analysis of different tumors, tumor microenvironment and tumor immune infiltration analysis, tumor immunotherapy analysis, and KEGG analysis. The results of this study demonstrated for the first time the expression and clinical significance of PKNOX1 in pan-cancer and its correlation with tumor immunity. Moreover, a series of experiments were performed to reveal, for the first time, the role of PKNOX1 in the occurrence and development of HCC and breast cancer.

In this study, we analysed the expression pattern of PKNOX1 in pan-cancer via the TCGA and GTEx databases. PKNOX1 was highly expressed in 22 tumor tissues, including BRCA, CHOL, LIHC, LUAD, LUSC, STAD, COAD, ACC, and SKCM, which was consistent with the reported results for gastric adenocarcinoma and non-small cell carcinoma (17, 18) and with the results of our immunohistochemical analysis in liver cancer, lung cancer, gastric cancer, and intestinal cancer. Moreover, compared with patients with low PKNOX1 expression, patients with high PKNOX1 expression had significantly worse OS in ACC, BRCA, ESCA, GBMLGG, KIRP, LUAD, LGG, LIHC, MESO, OV, SARC, STAD, STES, THCA, UCEC and UVM, but significantly correlated with better OS in BLCA, KIRC, READ and SKCM. Interestingly, PKNOX1 is highly expressed in SKCM, but high PKNOX1 expression was not a protective factor agains SKCM according to univariate Cox analysis. This contradiction may be related to the large difference in sample size for OS analysis. We also observed that low expression of PKNOX1 in KIRC was significantly associated with poor OS, DSS and PFI, which was similar to the single-factor Cox regression results. In terms of the expression pattern, we also observed a trend toward low expression of PKNOX1 in KIRC. We speculate that low expression of PKNOX1 in KIRC is related to tumorigenesis, and relevant reports have been published previously (28, 29). In LIHC, PKNOX1 is highly expressed and significantly associated with poor OS, DSS, and PFI. Univariate Cox analysis also revealed that high PKNOX1 expression is a significant risk factor for OS, DSS, and PFI. Our analysis of clinical characteristics revealed that high expression of PKNOX1 is significantly associated with increased clinical and pathological stages of LIHC. In addition, we conducted WB experiments to verify that PKNOX1 is highly expressed in both HCC cells and tissues. Consistent results were obtained in breast cancer validation.These findings are consistent with the results of this analysis. Thus, PKNOX1 may be an effective prognostic marker for cancers. In conclusion, our results suggest that PKNOX1 has different prognoses in different tumor types, is highly expressed in most tumors and is associated with poor prognosis, and PKNOX1 may serve as a promising prognostic marker in patients with ACC, GBMLGG, KIRP, LGG, LIHC, MESO, SARC, UVM, OV, etc.

Abnormal gene expression caused by gene mutation is a common feature of tumor cells and is closely related to the occurrence, development and metastasis of tumors. Genomic mutations and DNA methylation can lead to genomic stability disorders and induce the occurrence and development of tumors (21, 30–33). This study revealed that the frequency of PKNOX1 gene mutation was highest in patients with gastric adenocarcinoma. Previous studies have shown that PKNOX1 can promote the progression of gastric adenocarcinoma by regulating the Hedgehog signalling pathway (17), and the results are consistent. PKNOX1 is mainly “mutated” and “amplified” in tumors, and is concentrated mainly in tumors such as uterine endometrial cancer, acute myeloid leukemia, and gastric adenocarcinoma. We also found that PKNOX1 mRNA expression was significantly correlated with CNV in most tumors, including LUSC, OV, HNSC and other tumors. In addition, we also found that the methylation level of PKNOX1 was significantly negatively correlated with the expression levels of 19 tumor genes, including TGCT, STAD, LUSC, etc. We further analysed the correlation between PKNOX1 and nine methylation-related genes and found that PKNOX1 expression was more closely related to YTHDC1 and YTHDF3 expression and was significantly positively correlated in most tumors. These results reveal the potential reasons for the abnormal expression of PKNOX1 in tumors from the perspective of genetic variation and DNA modification. We speculate that PKNOX1 may promote tumorigenesis through DNA methylation, but further experimental research is required.

The TME includes cellular and non-cellular components, mainly immune cells, stromal cells and matrix components, and is involved in multiple biological processes such as tumor gene mutation expression, tumor occurrence, tumor development and metastasis, tumor immune escape and immunotherapy resistance (34). This study analysed the correlations between PKNOX1 and immune infiltration and immune cells in pan-cancer TME. MDSC is an important immune cell in tumors, that have immunosuppressive functions and can protect tumor cells from attack by the immune system (35). This study revealed that PKNOX1 expression was positively correlated with MDSC infiltration in most tumors. We speculate that PKNOX1 may be associated with tumor immunosuppression and immune escape. We further analysed the correlation between PKNOX1 and different types of immune cells and found that PKNOX1 was positively correlated with naive B cells, resting NK cells, neutrophils, etc., and negatively correlated with memory B cells, CD8+ T cells, activated NK cells, etc. These findings further indicate that PKNOX1 may be related to tumor immunosuppression. PKNOX1 may be able to protect tumor cells by inhibiting the body’s immunity and promoting tumor progression and metastasis. The regulation of the TME by genes is extremely complex. We need to further explore the regulatory mechanism between PKNOX1 and tumor immunity is needed to confirm this finding. In the future, PKNOX1 could be used as a new target and direction for exploring tumor immunotherapy drugs.

ICIs, which are emerging therapeutic drugs for treating tumors in recent years, have shown considerable therapeutic benefits and are promissing for many patients (36). However, in clinical applications, only a small number of patients can benefit from a single ICI in the long term and improve their survival, which reveals many problems in the application of ICIs, such as immunotoxic reactions, differences in the body’s immune response, and a lack of effective markers for predicting immune efficacy, seriously hindering the application of ICIs (20, 37–39). Therefore, it is highly important for the development of immunotherapies to explore effective immune biomarkers and determine which groups will benefit from ICIs treatment. TMB, MSI and PD-L1 are common markers related to tumor immunotherapy. There is no significant correlation between the two parameters in most tumors, and they are independent of each other (19, 40). This study conducted a comprehensive correlation analysis between PKNOX1 expression and current biomarkers of the response to ICIs (immune-related genes, TMB, and MSI). We found that PKNOX1 was significantly associated with immune-related genes in most tumors. PKNOX1 expression is significantly positively correlated with TMB and MSI in most tumors, and a negative correlation is observed in tumors such as BRCA and KIRP. These findings indicate that PKNOX1 has the potential to predict the efficacy of ICIs in certain tumors and may be used as an effective predictor.

Correlation analysis of PKNOX1 protein interactions and related genes revealed that the expression of GMEB1 was significantly positively correlated with that of PKNOX1. Glucocorticoid modulatoryelement-binding protein 1(GMEB1) has been confirmed to promote the occurrence and development of tumors. GMEB1 can promote the malignant proliferation and metastasis of HCC by promoting the transcription of the YAP1 promoter region (41); GMEB1 can also interact with USP40 to stabilize CFLARL in non-small cell lung cancer and inhibit cell apoptosis (42). We speculate that the interaction between PKNOX1 and GMEBA may also promote tumor progression. We further explored the function of PKNOX1 in tumors through KEGG pathway enrichment analysis and revealed that PKNOX1 was involved mainly in the cell cycle and the ERBB, NOTCH and WNT signalling pathways, which further indicated that PKNOX1 was closely related to tumor proliferation and progression.

HCC is the sixth most common malignant tumor and the third most fatal malignant tumor in the world (1). Our previous analysis also revealed that PKNOX1 is highly expressed in HCC and is closely related to poor patient prognosis and tumor immunity. In this study, we explored the biological function of PKNOX1 in HCC. The downregulation of PKNOX1 significantly affects the proliferation, growth, migration and invasion capabilities of HCC cells. The same results were further validated in breast cancer, another common tumor type.These results provide new evidence of the oncogenic function of PKNOX1 in cancers. Notably, we have only preliminarily explored the carcinogenic ability of PKNOX1 in HCC and breast cancer by combining online analysis databases and experiments. We need to further explore the specific oncogenic and cancer-promoting molecular mechanisms of PKNOX1 in HCC and breast cancer and even other tumors by combining relevant experiments and multiomics technology.

In general, this study is the first to investigate PKNOX1 in pan-cancer, and elaborates on the relevant functions of PKNOX1 at multiple levels, such as expression, gene mutation, DNA modification, survival prognosis, immune analysis, and enrichment analysis. PKNOX1 is highly expressed in most tumors and is associated with poor prognosis. Moreover, PKNOX1 is closely related to tumor immunity and treatment, and can be used as a biomarker for pan-cancer prognosis and immune efficacy prediction. This study also provides a theoretical basis for the development of PKNOX1 antitumor and immunotherapy targets. This study has certain limitations, such as only preliminary experimental verification for HCC and breast cancer; HCC and breast cancer experiments are limited to *in vitro* cell experiments, and no *in vivo* experiments have been conducted; related molecular mechanisms have not been explored in depth. In subsequent studies, further exploration of the detailed cancer-promoting mechanism of PKNOX1 is necessary.

## Conclusion

In summary, the results of this study indicate that PKNOX1 is highly expressed in most tumors and is closely related to poor prognosis, tumor immunity and therapy, and tumor proliferation and migration, providing a theoretical basis for the use of PKNOX1 as a pan-cancer prognostic biomarker and a tumor immunity biomarker.

## Data Availability

The original contributions presented in the study are included in the article/**Supplementary Material**. Further inquiries can be directed to the corresponding author.
