# Supplementary material for: Systematic pan-cancer analysis identifies PKNOX1 as a potential prognostic and immunological biomarker and its functional validation
Source: Front Immunol. 2025 Jun 23;16:1533690. doi: 10.3389/fimmu.2025.1533690 (PMC12230041; doi:10.3389/fimmu.2025.1533690)
Supplement: Supplementary file 1 [file DataSheet1.docx]

**Supplementary Table S1**  siRNA(PKNOX1) primer sequences

| **Primer Name** | **Sequence (5'to3')** |
| --- | --- |
| PKNOX1（human）siRNA-#1 | GCGGGCUGGUCUUGGAGAATT |
|  | UUCUCCAAGACCAGCCCGCTT |
| PKNOX1（human）siRNA-#2 | GGUUAACGAACUCUGCAAATT |
|  | UUUGCAGAGUUCGUUAACCTT |
| PKNOX1（human）siRNA-#3 | CCCAACAGAGGAUGAGAAATT |
|  | UUUCUCAUCCUCUGUUGGGTT |
| si-NC | UUCUCCGAACGUGUCACGUTT |
|  | ACGUGACACGUUCGGAGAATT |


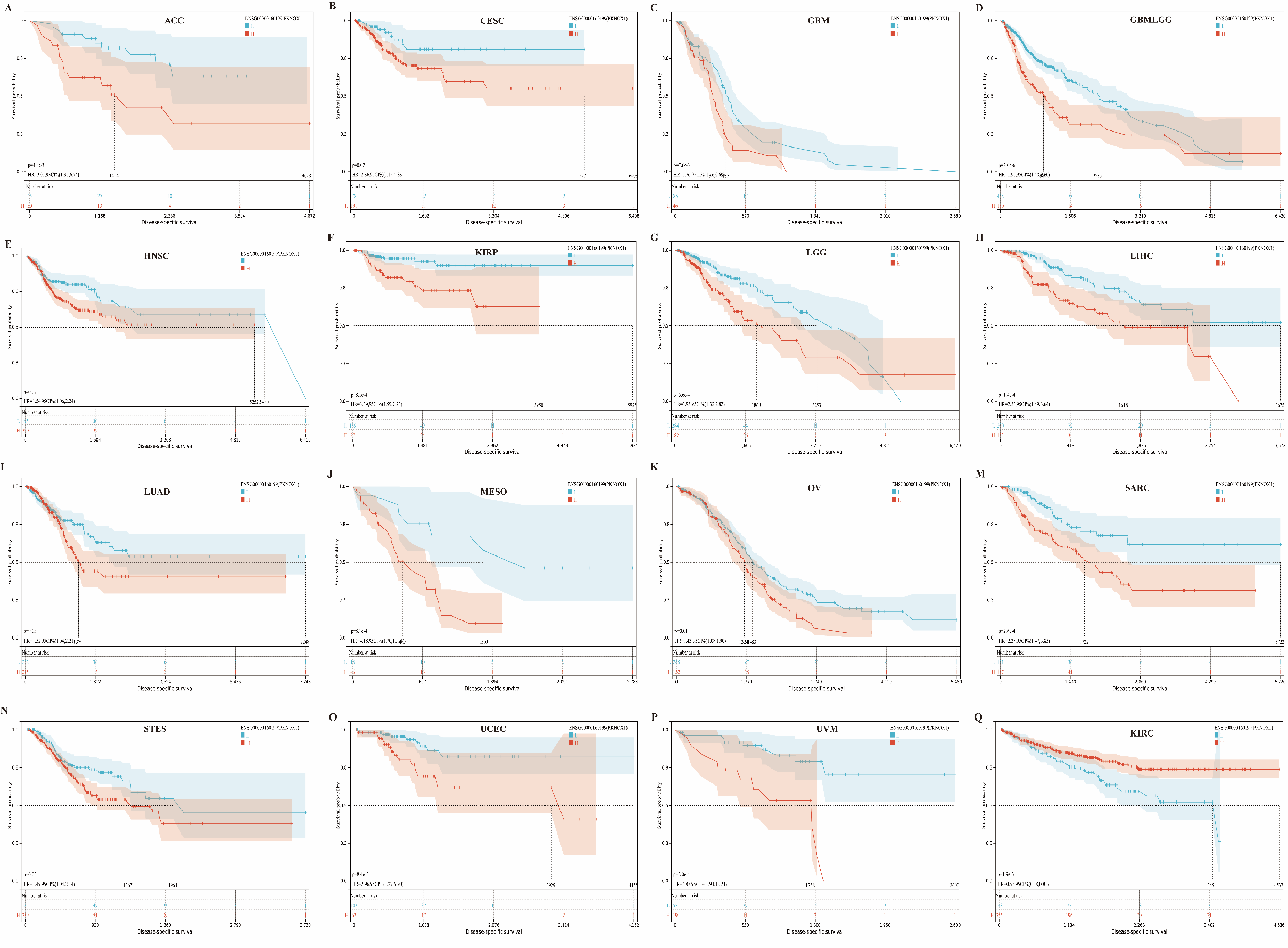


**Supplementary Figure S1** PKNOX1 expression was significantly associated with disease-specific survival (DSS) in 16 tumors according to Kaplan-Meier analysis. （A）ACC（B）CESC（C）GBM（D）GBMLGG（E）HNSC（F）KIRP（G）LGG（H）LIHC（I）LUAD（J）MESO（K）OV（M）SARC（N）STES（O）UCEC（P）UVM（Q）KIRC.


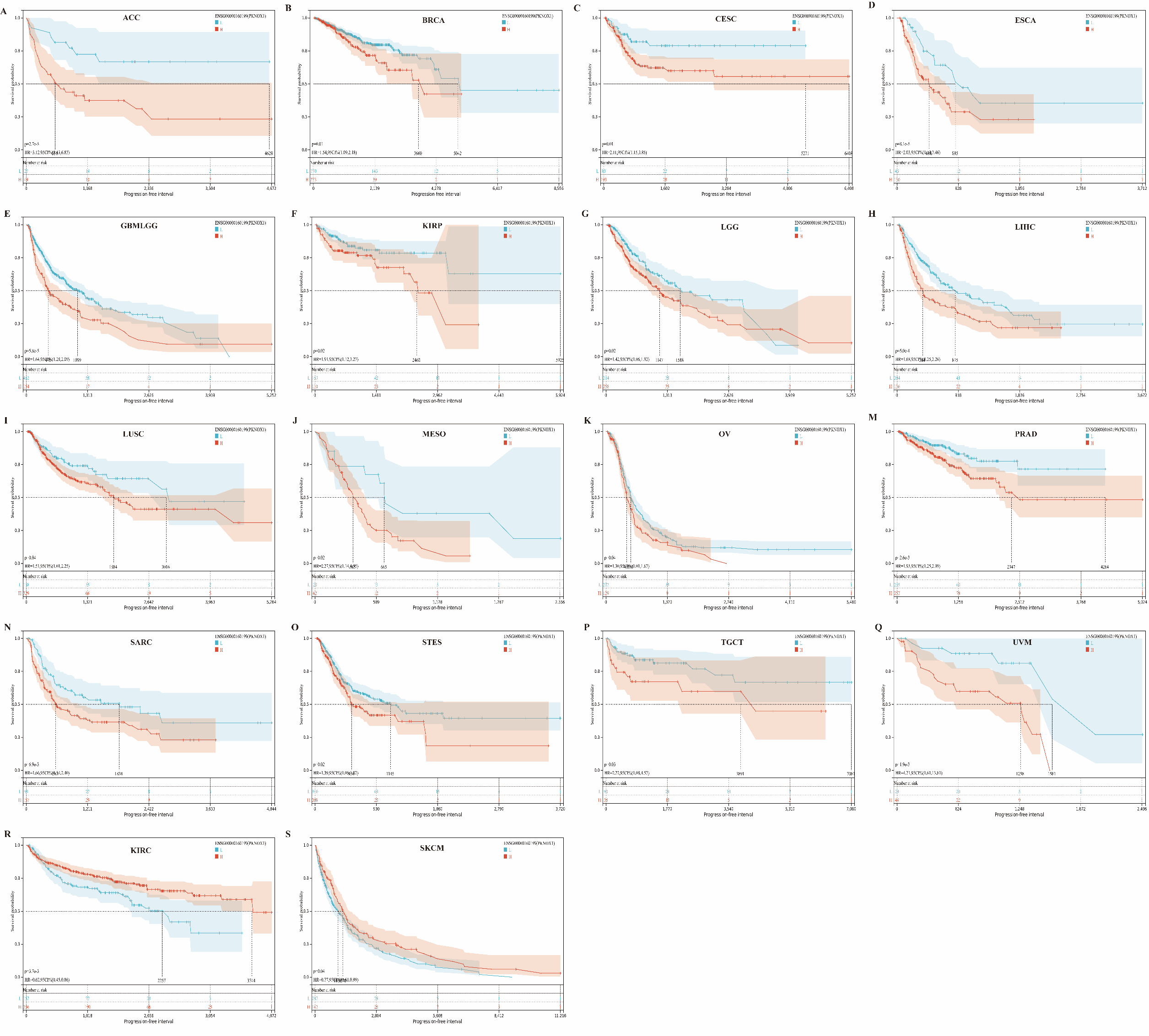


**Supplementary Figure S2** PKNOX1 expression was significantly associated with progression-free interval (PFI) in 18 tumors according to Kaplan-Meier analysis.（A）ACC（B）BRCA（C）CESC（D）ESCA（E）GBMLGG（F）KIRP（G）LGG（H）LIHC（I）LUSC（J）MESO（K）OV（M）PRAD（N）SARC（O）STES（P）TGCT（Q）UVM（R）KIRC（S）SKCM.
